# Methodological and statistical considerations in alcohol policy evaluation

**DOI:** 10.2471/BLT.14.151803

**Published:** 2015-08-05

**Authors:** Natacha Carragher, Joshua Byrnes, Christopher M Doran, Anthony Shakeshaft

**Affiliations:** a National Drug and Alcohol Research Centre, University of New South Wales, Sydney, New South Wales, Australia.; b Centre for Applied Health Economics, Griffith University, Meadowbrook, Australia.; c Hunter Medical Research Institute, New Lambton Heights, Australia.

Many countries recognize the adverse public health consequences of excessive alcohol consumption and have introduced alcohol preventive policies, such as a minimum age for alcohol purchase.^1^ However, few tools exist that quantitatively assess the level of stringency and enforcement of alcohol policies. Our development of the Toolkit for Evaluating Alcohol policy Stringency and Enforcement (TEASE-16) aimed to address this.

We applied the toolkit to nine study areas in the western Pacific. While this involved extensive data collection (288 data points) to develop the policy scores, as we noted in our paper,^2^ the small number of study areas under investigation reduced statistical power and, by default, restricted the choice of analytic method. The use of multiple regression analysis, as suggested by Duffy,^3^ is therefore substantially underpowered^4^ (*n* = 8 in the analysis presented by Duffy) and, in our paper, we cautioned against extrapolating the data for this reason. Future studies using the TEASE-16 to examine policy data from a larger number of countries will be better placed to conduct multiple regression analysis and account for the relationship between policy and consumption and alcohol-related harms. We also hope that future studies using the TEASE-16 will collect policy data over time to facilitate longitudinal regression analysis and tests of causality.

We used a univariate linear regression model to examine the relationship between alcohol policy and consumption. However, we also suggested that this relationship may not be linear and is likely to be dynamic over time. Exploratory analyses indicated that a log transformation of the policy score may improve regression fit, (as measured by the R^2^), suggesting that increases in policy scores would exert a greater impact on consumption for study areas with weak policy frameworks than for study areas with strong policy frameworks.

**Fig. 1:**
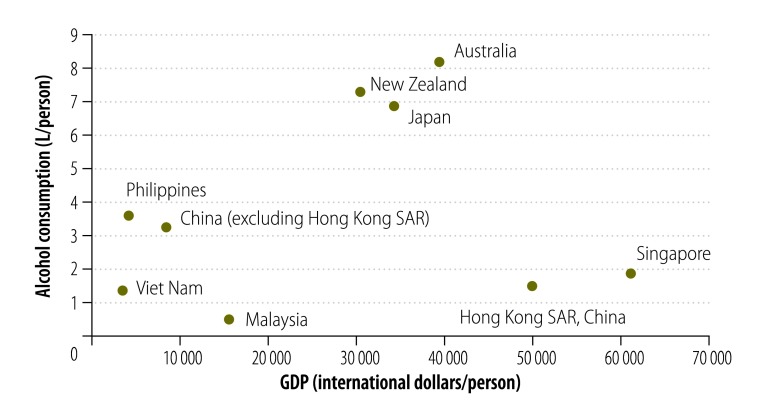
Relationship between GDP and alcohol consumption in the western Pacific

GDP: gross domestic product; L: litre; SAR: special administrative region.

Duffy queries our decision to adjust for income in per capita consumption estimates. We applied the TEASE-16 to a range of economically diverse study areas in the western Pacific. Based on economic theory and empirical studies, income is a significant determinant of consumption.^5^ Specifically, we included income in the analysis as litres of pure alcohol per 1000 international dollars of gross domestic product (GDP) per capita. Expressing consumption per dollar of GDP is an appropriate presentation of evidence and is used in cross-country and time-series analysis. A common example is with respect to energy consumption.^6^ Further, our analysis appropriately uses international dollars which adjust for differences in the relative price and purchasing power, facilitating meaningful cross-national comparisons. Further, and in congruence with previous empirical results, we find that higher income study areas, included in the original analysis, are associated with higher alcohol consumption with two notable outliers: China, Hong Kong Special Administrative Region and Singapore (Fig. 1). It seems unlikely that any substantive analysis of alcohol consumption and income would differ from the strong theoretical and empirical relationship that increases in income are associated with increases in consumption.

In summary, the TEASE-16 provides a useful practical and empirical tool for quantifying the level of stringency and enforcement of alcohol policies. Further research, however, is needed to realize its potential. Future studies applying the tool to a larger number of countries and across time will provide critical insights into the nature of the relationship between alcohol policies, consumption and alcohol-related harms.
